# Using an Ontology to Facilitate More Accurate Coding of Social Prescriptions Addressing Social Determinants of Health: Feasibility Study

**DOI:** 10.2196/23721

**Published:** 2020-12-11

**Authors:** Anant Jani, Harshana Liyanage, Cecilia Okusi, Julian Sherlock, Uy Hoang, Filipa Ferreira, Ivelina Yonova, Simon de Lusignan

**Affiliations:** 1 Oxford Martin School University of Oxford Oxford United Kingdom; 2 Department of Primary Care Health Sciences University of Oxford Oxford United Kingdom

**Keywords:** social prescribing, clinical informatics, ontology, social determinants of health

## Abstract

**Background:**

National Health Service (NHS) England supports social prescribing in order to address social determinants of health, which account for approximately 80% of all health outcomes. Nevertheless, data on ongoing social prescribing activities are lacking. Although NHS England has attempted to overcome this problem by recommending 3 standardized primary care codes, these codes do not capture the social prescribing activity to a level of granularity that would allow for fair attribution of outcomes to social prescribing.

**Objective:**

In this study, we explored whether an alternative approach to coding social prescribing activity, specifically through a social prescribing ontology, can be used to capture the social prescriptions used in primary care in greater detail.

**Methods:**

The social prescribing ontology, implemented according to the Web Ontology Language, was designed to cover several key concepts encompassing social determinants of health. Readv2 and Clinical Terms Version 3 codes were identified using the NHS Terms Browser. The Royal College of General Practitioners Research Surveillance Centre, a sentinel network of over 1000 primary care practices across England covering a population of more than 4,000,000 registered patients, was used for data analyses for a defined period (ie, January 2011 to December 2019).

**Results:**

In all, 668 codes capturing social prescriptions addressing different social determinants of health were identified for the social prescribing ontology. For the study period, social prescribing ontology codes were used 5,504,037 times by primary care practices of the Royal College of General Practitioners Research Surveillance Centre as compared to 29,606 instances of use of social prescribing codes, including NHS England’s recommended codes.

**Conclusions:**

A social prescribing ontology provides a powerful alternative to the codes currently recommended by NHS England to capture detailed social prescribing activity in England. The more detailed information thus obtained will allow for explorations about whether outputs or outcomes of care delivery can be attributed to social prescriptions, which is essential for demonstrating the overall value that social prescribing can deliver to the NHS and health care systems.

## Introduction

Approximately 80% of health outcomes are linked to social determinants of health, which include health-related behaviors as well as socioeconomic and environmental factors [1,2]. Social prescribing is a relatively recent initiative that has been developed to address the social determinants of health. National Health Service (NHS) England defines social prescribing as “a way of linking patients in primary care with sources of support within the community to help improve their health and wellbeing” [3]. Social prescriptions are varied and are mostly delivered by voluntary, community, and social enterprise (VCSE) organizations. The activities delivered by VCSEs range from health (eg, local walking groups), education (eg, dietary classes), skills development (eg, to facilitate employment), sports (eg, parkrun), and leisure or art (eg, singing groups) activities [4].

Despite its promise, a major barrier to the evaluation of social prescribing is the lack of data on what social prescribing activity is taking place and the outcomes delivered for people participating in these activities. This stems from the lack of information on the prescribed social prescriptions as well as variation in the quality of data recorded by clinicians [5].

In an attempt to address these gaps, NHS England worked with commissioners, practitioners, providers, evaluators, and other stakeholder groups to create a consensus Common Outcomes Framework (COF) on the outcomes and outputs that should be measured to demonstrate the impact of social prescribing. NHS England published the COF in 2019 [3] and recommended the use of 3 primary care codes to standardize the recording of social prescribing activity in primary care: “social prescribing offered,” “social prescribing declined,” and “referral to social prescribing service,” which are characterized as “finding’,” “situation,” and ”procedure,” respectively, in the Systematized Nomenclature of Medicine–Clinical Terms (SNOMED CT) concept top-level hierarchy [6].

A standardization of how social prescriptions are recorded in primary care is essential to improve data quality so the general approach advocated by NHS England with the COF is sound. However, the codes recommended by NHS England have several limitations, which stem from their very general nature. For instance, the corresponding equivalent codes for pharmaceutical prescriptions would be “pharmaceutical prescription offered,” ”pharmaceutical prescription declined,” and ”pharmaceutical prescription given.” The general nature of these codes means that they do not capture the actual intervention delivered, which means that we cannot extrapolate which outcomes could realistically have been delivered by the social prescription; therefore, we cannot accurately attribute any outcomes to the actual social prescription. These limitations imply that if we only rely on these codes, it would be impossible to know whether social prescriptions deliver any benefit.

In this study, we explored whether an alternative approach to coding social prescribing activity can be used to capture more detail on the actual social prescriptions used. Specifically, we used well-established ontological approaches, which are used for modeling the semantics of medical concepts [7], to explore whether:

- a social prescribing ontology can be created with existing primary care codes to capture more detail on which social prescriptions are prescribed by primary care professionals
- the ontological codes are actually used by primary care professionals in practice
- a social prescribing ontology can serve as a viable alternative to capture more detailed information on social prescriptions in England

## Methods

The study methods were essentially the same as previously reported [8] but are discussed briefly below.

### Designing and Compiling the Ontology

**Figure 1:**
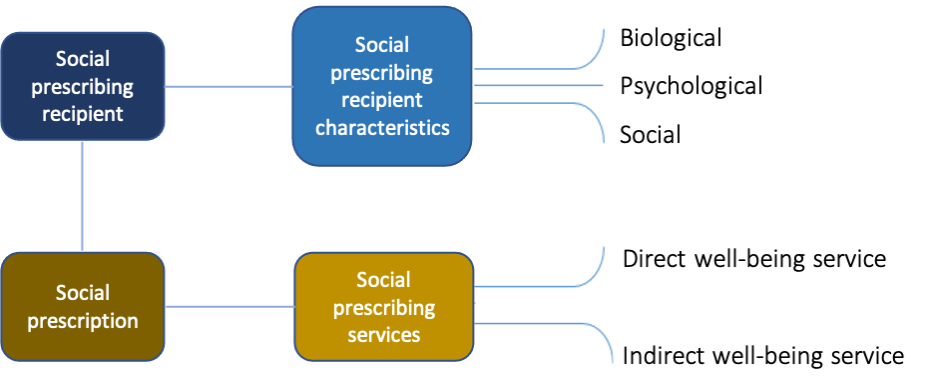
Design of the social prescribing ontology.

An ontology is defined as a set of concepts and categories in a subject area or domain that describes their properties and the relations between them. The social prescribing ontology covers several key concepts derived from the “Five Ways to Wellbeing” model proposed by the New Economics Foundation [9] as well as Wilkinson and Marmot’s work [2] on social determinants of health (Figure 1 and Table 1).

**Table 1:** Social prescribing ontological categories and unique primary codes (N=668) for each category.

Social prescribing ontological category	Unique primary care codes, n
Addictions support services	35
Benefits signposting services	10
Bereavement support services	20
Dementia support services	13
Diabetes management support services	11
Dietary support services	185
Domestic violence support services	2
Education support services	1
employment support services	20
finance support services	4
General lifestyle support services	15
General social support services	27
Home-based support services	19
Housing support services	25
Mental health services	17
Support services for other conditions	21
Parental support services	139
Physical activity management services	89
Respiratory support services	6
Stress reduction support services	9

The Readv2 and Clinical Terms Version 3 (CTV3) codes that comprise the social prescribing ontology were identified through 2 NHS Digital resources: (1) the NHS Term Browser, which is hosted by NHS Digital to provide a means to browse and search the SNOMED CT UK Edition, and (2) the Readv2 CTV3 to SNOMED CT Mapping Lookup, which maps SNOMED CT to the Readv2 and CTV3 terminologies. The social prescribing ontology has been implemented according to the Web Ontology Language (OWL) within the Protégé ontology development environment and hosted on the BioPortal ontology repository [10].

### Data Analysis

We utilized the Royal College of General Practitioners Research Surveillance Centre (RCGP RSC) sentinel network as previously described [8]. The RCGP RSC was established in 1967 and comprises computerized medical record (CMRs) of pseudonymized data received from over 1000 primary care practices across England, covering a population of more than 4,000,000 currently registered patients [11,12].

CMR data in UK primary care centers are captured primarily within 2 electronic health record (EHR) systems that utilize Readv2 and CTV3 codes. Both these systems will be transitioning to SNOMED CT, but the analyses in this study relied on historical data from 2011 to 2019 so we did not use SNOMED CT codes in the data extracts. Readv2 and CTV3 codes are used to collate data for primary care, including diagnoses, processes of care, prescriptions, and results from laboratory-based data.

We extracted and analyzed coded, pseudonymized data from the RCGP RSC sentinel network primary care practices from January 1, 2011, to December 31, 2019. The data extracts included all instances of use of the codes highlighted in Supplementary Table S1 (Multimedia Appendix 1).

### Ethical Approval

Consent was not required for the RCGP RSC data. Furthermore, data were not processed for individuals who had active opt-out codes present (which comprises 2.74% of registered patients as of March 7, 2019) [13]. The data were pseudonymized and encrypted before they were uploaded to the Clinical Informatics Research Group secure server. Personal data was not identifiable. This study was considered to be an “audit of current practice” when tested against the Health Research Authority/Medical Research Council “Is my study research” tool [14] and, therefore, did not require specific ethical approval. The RCGP RSC Study Approval Committee approved the use of data.

Data extractions were conducted in accordance with the Clinical Informatics and Health Outcomes Research Group’s standard operating procedures for data extraction, pseudonymization, and transfer, as described previously [15].

## Results

### Social Prescribing Ontology

Twenty ontological categories were identified with a total of 668 codes heterogeneously distributed across all ontological categories, ranging from 185 codes for “Dietary support services” to only 1 code for “Education support services” (see Table 1 and Supplementary Table S1 in Multimedia Appendix 1).

### Determining the Utilization of Social Prescribing Ontological Codes

The RCGP RSC dataset was searched from January 01, 2011 to December 31, 2019, to determine the extent to which codes within the social prescribing ontology were used by RCGP RSC primary care practices in England. Codes for “social prescribing,” including the 3 codes recommended in the NHS England COF, were also investigated (for the full code list, see Supplementary Table S1 in Multimedia Appendix 1).

In all, 29,606 instances of use of “social prescribing” codes were found during the search period, compared to 5,504,037 instances of use of social prescribing ontology codes by RCGP RSC primary care practices (Table 2).

**Table 2:** Number of instances of use of social prescribing and social prescribing ontology codes within the Royal College of General Practitioners Research Surveillance Centre from January 01, 2011, to Dec 31, 2019 (N=5,533,643).

Category		Instances of code use recorded during the study period, n
**Social prescribing codes**		29,606
**Social prescribing ontology code**		5,504,037
	Dietary support services	2,087,171
	Physical activity management services	1,782,267
	Addictions support services	769,860
	General lifestyle support services	552,677
	Parental support services	94,766
	General social support services	75,321
	Diabetes management support services	73,404
	Homebased support services	22,198
	Bereavement support services	16,212
	Respiratory support services	9699
	Support services for other conditions	7400
	Mental health services	4868
	Dementia support services	3710
	Benefits signposting services	2169
	Stress reduction support services	1012
	Employment support services	743
	Housing support services	554
	Finances support services	6
	Domestic violence support services	0
	Education support services	0

## Discussion

In this study, we found that a social prescribing ontology could be used to provide more details about the type of social prescription utilized by primary care practices in England. We identified 668 existing codes within Readv2 and CTV3 code sets that captured social prescriptions to a greater level of detail than those captured by the recommended NHS England codes of “social prescribing offered,” “social prescribing declined,” and “referral to social prescribing service.” We also found that the ontology codes were regularly used by primary care professionals across the nationally representative RCGP RSC sentinel network with over 5 million instances of use recorded between January 2011 and December 2019.

Our study demonstrates that primary care professionals have been regularly using the codes identified within our social prescribing ontology since 2011. This finding indicates these professionals were already aware of these codes and were using nonmedical interventions to address the social needs of patients through their existing primary care workforce, that is, before the establishment of link workers. With support from NHS England and key stakeholders, a social prescribing ontology could be recommended from a policy perspective, and it could be used nationally to improve data quality on social prescribing. Creating a national social prescribing ontology will be an iterative process that will require engagement with key stakeholders and consensus building—similar to the process used to create the COF. This process will also help clarify what can be truly characterized as a social prescription because some interventions such as education are not limited to only social prescribing, and this can ultimately inform the creation of new codes within SNOMED CT. Furthermore, given that the codes used for the ontology already exist in primary care code sets, templates could be created in primary care EHRs to facilitate access and utilization of these codes to more accurately capture social prescribing activity while also creating the digital infrastructure needed to create a social prescribing formulary [16].

Our study findings demonstrate that a social prescribing ontology, if appropriately designed, provides a powerful alternative to the codes currently recommended by NHS England to capture social prescribing activity. This is because such an ontology provides more granular information on the actual social prescription used, which will allow for explorations about whether outputs or outcomes of care delivery can be attributed to social prescriptions. These are essential steps for demonstrating the overall value that social prescribing can deliver to the NHS and health care systems.

## Appendix

Multimedia Appendix 1 Social prescribing ontology code list.

## Abbreviations

CMR: computerized medical record
COF: Common Outcomes Framework
CTV3: Clinical Terms Version 3
EHR: electronic health record
NHS: National Health Service
OWL: Web Ontology Language
RCGP RSC: Royal College of General Practitioners Research Surveillance Centre
SNOMED CT: Systematized Nomenclature of Medicine–Clinical Terms
VCSE: voluntary, community and social enterprise
