# Supplementary material for: Using an Ontology to Facilitate More Accurate Coding of Social Prescriptions Addressing Social Determinants of Health: Feasibility Study
Source: J Med Internet Res. 2020 Dec 11;22(12):e23721. doi: 10.2196/23721 (PMC7762682; doi:10.2196/23721)
Supplement: Multimedia Appendix 1 [file jmir_v22i12e23721_app1.docx]

*Supplementary Table 1***.** Social prescribing ontology code list.

| **Count** | **Category** | **Coding System** | **Code** | **Term** |
| --- | --- | --- | --- | --- |
| 35 | Addictions Support Services | CTV3 | .13Y8 | Alcoholics anonymous |
|  | Addictions Support Services | CTV3 | 6792. | Health ed. - alcohol |
|  | Addictions Support Services | CTV3 | 679A. | Health ed.- drugs of addiction |
|  | Addictions Support Services | CTV3 | 67H3. | Lifestyle adv re drug misuse |
|  | Addictions Support Services | CTV3 | 8BAX. | Drug depen home detoxification |
|  | Addictions Support Services | CTV3 | XaJIu | Lifestyle adv re drug misuse |
|  | Addictions Support Services | CTV3 | .6791 | Health education - smoking |
|  | Addictions Support Services | CTV3 | .6792 | Health education - alcohol |
|  | Addictions Support Services | CTV3 | .8BAX | Drug depen home detoxification |
|  | Addictions Support Services | CTV3 | 6791. | Health education - smoking |
|  | Addictions Support Services | CTV3 | 6792. | Health education - alcohol |
|  | Addictions Support Services | CTV3 | 8BAx. | Drug twelve step programme |
|  | Addictions Support Services | CTV3 | Xa360 | Advice to change alcohol intak |
|  | Addictions Support Services | CTV3 | XaIOs | Ment healt addiction programm |
|  | Addictions Support Services | CTV3 | XaIWd | Mental health addiction programmes - 24 hour not intensive |
|  | Addictions Support Services | CTV3 | XaIWe | Mental health addiction programmes - 24 hour intensive |
|  | Addictions Support Services | CTV3 | XaIWf | Mental health addiction programmes - full day : day care |
|  | Addictions Support Services | CTV3 | XaIWg | Mental health addiction programmes - part day : day care |
|  | Addictions Support Services | CTV3 | XaIWh | Mental health addiction programmes - daily intensive |
|  | Addictions Support Services | CTV3 | XaIWi | Mental health addiction programmes - 3-5 contacts/week |
|  | Addictions Support Services | CTV3 | XaIWj | Mental health addiction programmes - 1-2 contacts/week |
|  | Addictions Support Services | CTV3 | XaIWk | Mental health addiction programmes - 1-3 contacts/month |
|  | Addictions Support Services | CTV3 | XaIWl | Mental health addiction programmes - <1 contact/month |
|  | Addictions Support Services | CTV3 | XaLsw | Delivery rehab drug addiction |
|  | Addictions Support Services | CTV3 | XaLsx | Delivery of rehabilitation for alcohol addiction |
|  | Addictions Support Services | CTV3 | XE1TQ | Advice on drugs of addiction |
|  | Addictions Support Services | CTV3 | ZV6D7 | [V]Drug abuse counsel+surveiln |
|  | Addictions Support Services | Readv2 | 13Y8. | Alcoholics anonymous |
|  | Addictions Support Services | Readv2 | 6792. | Health ed. - alcohol |
|  | Addictions Support Services | Readv2 | 7P220 | Delivery rehab drug addiction |
|  | Addictions Support Services | Readv2 | 7P221 | Del rehab alcohol addiction |
|  | Addictions Support Services | Readv2 | 8B23. | Drug addiction therapy |
|  | Addictions Support Services | Readv2 | 8BAX. | Drug depen home detoxification |
|  | Addictions Support Services | Readv2 | 8BAx. | Drug twelve step programme |
|  | Addictions Support Services | Readv2 | ZV6D7 | [V]Drug abuse counsel+surveiln |
| 10 | Benefits Signposting Services | CTV3 | 13OY. | Benefits - other, specified |
|  | Benefits Signposting Services | CTV3 | 13VP. | Benefits received |
|  | Benefits Signposting Services | CTV3 | 6743. | Benefits counselling |
|  | Benefits Signposting Services | CTV3 | 9EY3. | Report to benefits agency |
|  | Benefits Signposting Services | CTV3 | 9EY4. | Report request benefits agency |
|  | Benefits Signposting Services | CTV3 | Ua0LC | Not receiving all benefits due |
|  | Benefits Signposting Services | CTV3 | Ua0LD | Delay in receiving benefits |
|  | Benefits Signposting Services | CTV3 | Ua0mc | Local authority benefits |
|  | Benefits Signposting Services | CTV3 | Ua0mS | National Hlth Service benefits |
|  | Benefits Signposting Services | CTV3 | XaKyh | Referral to benefits officer |
| 20 | Bereavement Support Services | CTV3 | 13Q.. | Widows benefits |
|  | Bereavement Support Services | CTV3 | 13QZ. | Widows benfits NOS |
|  | Bereavement Support Services | CTV3 | XaIpF | Bereavement support |
|  | Bereavement Support Services | Readv2 | 6751 | Bereavement counselling |
|  | Bereavement Support Services | Readv2 | 8HHK. | Referral to bereavement counsellor |
|  | Bereavement Support Services | CTV3 | .6751 | Bereavement counselling |
|  | Bereavement Support Services | CTV3 | .8HHK | Ref to bereavement counsellor |
|  | Bereavement Support Services | CTV3 | .8O81 | Bereavement support |
|  | Bereavement Support Services | CTV3 | 6751. | Bereavement counselling |
|  | Bereavement Support Services | CTV3 | 8HHK. | Ref to bereavement counsellor |
|  | Bereavement Support Services | CTV3 | 8O81. | Bereavement support |
|  | Bereavement Support Services | CTV3 | Ua1NY | Bereavement support visit |
|  | Bereavement Support Services | CTV3 | XaAfG | Ref to bereavement counsellor |
|  | Bereavement Support Services | CTV3 | XaAOe | Under care bereavement cllr |
|  | Bereavement Support Services | CTV3 | XaAS1 | Seen by bereavement counsellor |
|  | Bereavement Support Services | CTV3 | XaAZF | Ref from bereavemt counsellor |
|  | Bereavement Support Services | CTV3 | XafMP | Signposting Cruse Brvmnt Care |
|  | Bereavement Support Services | CTV3 | XagRU | Signpost to berevemnt supp srv |
|  | Bereavement Support Services | Readv2 | 6751. | Bereavement counselling |
|  | Bereavement Support Services | Readv2 | 8O81. | Bereavement support |
| 13 | Dementia Support Services | CTV3 | Xabd2 | Refl to demtia supprt org dcld |
|  | Dementia Support Services | CTV3 | Xabd3 | Refl to dementia support orgsn |
|  | Dementia Support Services | CTV3 | 8Hla. | Referral dementia care advisor |
|  | Dementia Support Services | CTV3 | 8T05. | Referral to dementia service |
|  | Dementia Support Services | CTV3 | 8T050 | Refl to dementia support orgsn |
|  | Dementia Support Services | CTV3 | 8T051 | Referral to dementia support organisation declined |
|  | Dementia Support Services | CTV3 | XagMZ | Signpost to dementia suprt srv |
|  | Dementia Support Services | CTV3 | XaYFR | Referral dementia care advisor |
|  | Dementia Support Services | CTV3 | XaZqJ | Referral to dementia service |
|  | Dementia Support Services | Readv2 | 8Hla. | Referral dementia care advisor |
|  | Dementia Support Services | Readv2 | 8T05. | Referral to dementia service |
|  | Dementia Support Services | Readv2 | 8T050 | Refl to dementia support orgsn |
|  | Dementia Support Services | Readv2 | 8T051 | Referral to dementia support organisation declined |
| 11 | Diabetes Management Support Services | CTV3 | .679L | Health education - diabetes |
|  | Diabetes Management Support Services | CTV3 | 679L0 | Educa self management diabetes |
|  | Diabetes Management Support Services | CTV3 | Xaafx | Educat abt lifestyl rsk diabet |
|  | Diabetes Management Support Services | CTV3 | XabZm | Lifestyle education diabetes |
|  | Diabetes Management Support Services | Readv2 | 8Hj4. | Referral to DESMOND structured programme (procedure) |
|  | Diabetes Management Support Services | Readv2 | 8I83. | Did not complete DESMOND structured programme (Situation) |
|  | Diabetes Management Support Services | Readv2 | 8IE9. | Referral to DESMOND structured programme declined (situation) |
|  | Diabetes Management Support Services | Readv2 | 9NiD. | Did not attend DESMOND structured programme (finding) |
|  | Diabetes Management Support Services | Readv2 | 9OLE. | Attended DESMOND programme (finding) |
|  | Diabetes Management Support Services | Readv2 | 9OLK. | DESMOND structured programme completed (situation) |
|  | Diabetes Management Support Services | Readv2 | 9OLP. | Expert patient education versus routine treatment First steps diabetes self management programme completed (situation) |
| 185 | Dietary Support Services | CTV3 | .67H7 | Lifestyle advice regard diet |
|  | Dietary Support Services | Readv2 | 66CR. | Intervention for risk to health associated with overweight and obesity, advice about diet and physical activity |
|  | Dietary Support Services | Readv2 | 6799 | Health ed. - diet |
|  | Dietary Support Services | Readv2 | 679Q. | Health education - nutrition |
|  | Dietary Support Services | Readv2 | 8CA4M | High energy diet education |
|  | Dietary Support Services | Readv2 | 8CA4N | Vitamin education |
|  | Dietary Support Services | Readv2 | 8CA4Q | Healthy eating education |
|  | Dietary Support Services | Readv2 | 8CA4V | Dietary education to maximise nutritional intake |
|  | Dietary Support Services | Readv2 | 8CJ5. | Nutritional therapy |
|  | Dietary Support Services | CTV3 | .6798 | Health education - exercise |
|  | Dietary Support Services | CTV3 | .6799 | Health education - diet |
|  | Dietary Support Services | CTV3 | .679Q | Health education - nutrition |
|  | Dietary Support Services | CTV3 | .8CA4 | Advice on diet |
|  | Dietary Support Services | CTV3 | .8CAR | Pt advised re low salt diet |
|  | Dietary Support Services | CTV3 | .8CE4 | Diet leaflet given |
|  | Dietary Support Services | CTV3 | .8CJ. | Nutrition |
|  | Dietary Support Services | CTV3 | .8H76 | Refer to dietician |
|  | Dietary Support Services | CTV3 | 66CS. | Intervention for risk to health associated with overweight and obesity, advice about diet and physical activity, consider drugs |
|  | Dietary Support Services | CTV3 | 66CT. | Intervention for risk to health associated with overweight and obesity, advice about diet and physical activity, consider drugs, consider surgery |
|  | Dietary Support Services | CTV3 | 6799. | Health education - diet |
|  | Dietary Support Services | CTV3 | 679Q. | Health education - nutrition |
|  | Dietary Support Services | CTV3 | 67A10 | Education about cow's milk protein free diet |
|  | Dietary Support Services | CTV3 | 67H7. | Lifestyle advice regard diet |
|  | Dietary Support Services | CTV3 | 8CA4. | Advice on diet |
|  | Dietary Support Services | CTV3 | 8CA40 | Pt advised re wt reducing diet |
|  | Dietary Support Services | CTV3 | 8CA42 | Pt advised re gluten free diet |
|  | Dietary Support Services | CTV3 | 8CA43 | Pt advised-phenylal. free diet |
|  | Dietary Support Services | CTV3 | 8CA44 | Pt advised re high fibre diet |
|  | Dietary Support Services | CTV3 | 8CA45 | Pt advised re low residue diet |
|  | Dietary Support Services | CTV3 | 8CA46 | Pt advised re low fat diet |
|  | Dietary Support Services | CTV3 | 8CA47 | Pt advis-low cholesterol diet |
|  | Dietary Support Services | CTV3 | 8CA48 | Pt advised re low salt diet |
|  | Dietary Support Services | CTV3 | 8CA49 | Pt advised - lactose free diet |
|  | Dietary Support Services | CTV3 | 8CA4A | Pt advis-low carbohydrate diet |
|  | Dietary Support Services | CTV3 | 8CA4a | Education about FODMAP (fermentable oligosaccharides, disaccharides, monosaccharides and polyols) exclusion diet |
|  | Dietary Support Services | CTV3 | 8CA4B | Pt advised re low protein diet |
|  | Dietary Support Services | CTV3 | 8CA4C | Pt advised - high protein diet |
|  | Dietary Support Services | CTV3 | 8CA4D | Pt advised re milk free diet |
|  | Dietary Support Services | CTV3 | 8CA4E | Pt advised re egg free diet |
|  | Dietary Support Services | CTV3 | 8CA4F | Pt advised re vegetarian diet |
|  | Dietary Support Services | CTV3 | 8CA4G | Pt advised re vegan diet |
|  | Dietary Support Services | CTV3 | 8CA4M | High energy diet education |
|  | Dietary Support Services | CTV3 | 8CA4N | Vitamin education |
|  | Dietary Support Services | CTV3 | 8CA4R | Fluid restriction advice |
|  | Dietary Support Services | CTV3 | 8CA4S | Dietary advice food allergy |
|  | Dietary Support Services | CTV3 | 8CA4V | Diet eductn max nutritn intake |
|  | Dietary Support Services | CTV3 | 8CA4z | Pt advised re diet NOS |
|  | Dietary Support Services | CTV3 | 8CE4. | Diet leaflet given |
|  | Dietary Support Services | CTV3 | 8CJ.. | Feeding and dietary regimes |
|  | Dietary Support Services | CTV3 | 8H76. | Refer to dietician |
|  | Dietary Support Services | CTV3 | 8H760 | Refer to comm-based dietitian |
|  | Dietary Support Services | CTV3 | 9N0H. | Seen in dietician clinic |
|  | Dietary Support Services | CTV3 | 9N27. | Seen by dietician |
|  | Dietary Support Services | CTV3 | 9NJ6. | In-house dietetics |
|  | Dietary Support Services | CTV3 | 9NJh. | In-house dietetics 1st appt |
|  | Dietary Support Services | CTV3 | 9NJj. | In-house dietetics f/up appt |
|  | Dietary Support Services | CTV3 | 9NNF. | Under care of dietitian |
|  | Dietary Support Services | CTV3 | Ub01h | Diet health promotion advice |
|  | Dietary Support Services | CTV3 | Ub01n | Diet intake assess procedures |
|  | Dietary Support Services | CTV3 | Ub01p | Review of current diet |
|  | Dietary Support Services | CTV3 | Ub01r | Diet int ass using food models |
|  | Dietary Support Services | CTV3 | Ub01s | Weighed dietary intake ass |
|  | Dietary Support Services | CTV3 | Ub01u | Diet in ass usng food fr quest |
|  | Dietary Support Services | CTV3 | Ub01w | Diet int ass using food photo |
|  | Dietary Support Services | CTV3 | Ub01y | Diets |
|  | Dietary Support Services | CTV3 | Ub01z | Modified diets |
|  | Dietary Support Services | CTV3 | Ub1md | Advice change dietary intake |
|  | Dietary Support Services | CTV3 | Ub1mg | Advice to change drink intake |
|  | Dietary Support Services | CTV3 | Ub1mi | Advic chang convenien food int |
|  | Dietary Support Services | CTV3 | Ub1qF | Advice change food add int |
|  | Dietary Support Services | CTV3 | Ub1u2 | Advice to change energy intake |
|  | Dietary Support Services | CTV3 | Xa0rr | Dietetic procedures |
|  | Dietary Support Services | CTV3 | Xa1dH | Pt advised re sugar-free diet |
|  | Dietary Support Services | CTV3 | Xa2jQ | Dietary advice for weight loss |
|  | Dietary Support Services | CTV3 | Xa2jT | Dietary advice for weight gain |
|  | Dietary Support Services | CTV3 | Xa2nG | Method of dietary advice given |
|  | Dietary Support Services | CTV3 | Xa2o4 | Advice to change nutritional p |
|  | Dietary Support Services | CTV3 | Xa2pA | Advice to change food preserva |
|  | Dietary Support Services | CTV3 | Xa35m | Advice change fat & oil intake |
|  | Dietary Support Services | CTV3 | Xa35n | Advice to change fish intake |
|  | Dietary Support Services | CTV3 | Xa35S | Advice to change cheese intake |
|  | Dietary Support Services | CTV3 | Xa35y | Advice change diet fibre intak |
|  | Dietary Support Services | CTV3 | Xa3BK | Advice change savoury food int |
|  | Dietary Support Services | CTV3 | Xa3BR | Advic change veg and pulse int |
|  | Dietary Support Services | CTV3 | Xa3BS | Advice to change veg intake |
|  | Dietary Support Services | CTV3 | Xa3BV | Advice to change potato intake |
|  | Dietary Support Services | CTV3 | Xa3BW | Advice change pulse veg intake |
|  | Dietary Support Services | CTV3 | Xa3BY | Advice change sav snack intak |
|  | Dietary Support Services | CTV3 | Xa3Fa | Advice change sugary food int |
|  | Dietary Support Services | CTV3 | Xa3Fb | Advice to change sugar intake |
|  | Dietary Support Services | CTV3 | Xa3Fp | Advice to change alcohol intak |
|  | Dietary Support Services | CTV3 | Xa3Fq | Advice to change coffee intake |
|  | Dietary Support Services | CTV3 | Xa3FR | Advice to change pudding intak |
|  | Dietary Support Services | CTV3 | Xa3FS | Advice change sandwich intake |
|  | Dietary Support Services | CTV3 | Xa3FU | Advice chang starch food intak |
|  | Dietary Support Services | CTV3 | Xa3FV | Advice to change biscuit intak |
|  | Dietary Support Services | CTV3 | Xa3FW | Advice to change bRead intake |
|  | Dietary Support Services | CTV3 | Xa3Fw | Advice change fizzy drink int |
|  | Dietary Support Services | CTV3 | Xa3FX | Advic chang brekfst cereal int |
|  | Dietary Support Services | CTV3 | Xa3Fx | Advice change fruit juice int |
|  | Dietary Support Services | CTV3 | Xa3FY | Advice to change pasta intake |
|  | Dietary Support Services | CTV3 | Xa3G2 | Advice change milk drink intak |
|  | Dietary Support Services | CTV3 | Xa3G4 | Advice to change tea intake |
|  | Dietary Support Services | CTV3 | Xa3G5 | Advice change carbo food intak |
|  | Dietary Support Services | CTV3 | Xa3G7 | Advice change fatty food intak |
|  | Dietary Support Services | CTV3 | Xa3G8 | Advic chang high ener food int |
|  | Dietary Support Services | CTV3 | Xa3G9 | Advic chang high fibr food int |
|  | Dietary Support Services | CTV3 | Xa3GA | Advice change low cal food int |
|  | Dietary Support Services | CTV3 | Xa3GC | Advic chang low fibre food int |
|  | Dietary Support Services | CTV3 | Xa3GF | Advice change red meat intake |
|  | Dietary Support Services | CTV3 | Xa3GG | Advice change white meat intak |
|  | Dietary Support Services | CTV3 | Xa3GH | Advice to change butter intake |
|  | Dietary Support Services | CTV3 | Xa3GI | Advice change margarine intake |
|  | Dietary Support Services | CTV3 | Xa3GJ | Advice to change ghee intake |
|  | Dietary Support Services | CTV3 | Xa3GK | Advice to change oil intake |
|  | Dietary Support Services | CTV3 | Xa3GL | Advice change cow's milk intak |
|  | Dietary Support Services | CTV3 | Xa3GP | Advice change soya milk intake |
|  | Dietary Support Services | CTV3 | Xa3GS | Advice change white fish intak |
|  | Dietary Support Services | CTV3 | Xa3GU | Advice change fatty fish intak |
|  | Dietary Support Services | CTV3 | Xa4Om | Advice change food colour int |
|  | Dietary Support Services | CTV3 | Xa97T | Feeding and dietary regimes |
|  | Dietary Support Services | CTV3 | Xaadb | High energy diet education |
|  | Dietary Support Services | CTV3 | Xaadc | Vitamin advice |
|  | Dietary Support Services | CTV3 | Xaadf | Fluid restriction advice |
|  | Dietary Support Services | CTV3 | XaAdX | Referral to dietetics service |
|  | Dietary Support Services | CTV3 | XaAdY | Refer to commun dietetics serv |
|  | Dietary Support Services | CTV3 | XaAdZ | Ref to hospital dietetics serv |
|  | Dietary Support Services | CTV3 | XaAha | Refer to hosp-based dietitian |
|  | Dietary Support Services | CTV3 | XaAhZ | Refer to comm-based dietitian |
|  | Dietary Support Services | CTV3 | XaARG | Under care of dietitian |
|  | Dietary Support Services | CTV3 | XaARH | Under care comm-based dietitn |
|  | Dietary Support Services | CTV3 | XaARI | Under care hosp-base dietitian |
|  | Dietary Support Services | CTV3 | Xaart | Diet eductn max nutritn intake |
|  | Dietary Support Services | CTV3 | XaAUb | Seen by commun-based dietitian |
|  | Dietary Support Services | CTV3 | XaAUc | Seen by hosp-based dietitian |
|  | Dietary Support Services | CTV3 | XaAX4 | Seen by dietetics - service |
|  | Dietary Support Services | CTV3 | XaAX5 | Seen by commun dietetic - serv |
|  | Dietary Support Services | CTV3 | XaAX6 | Seen by hosp dietetics - serv |
|  | Dietary Support Services | CTV3 | XaaxY | Dietetics intervention declind |
|  | Dietary Support Services | CTV3 | XabKZ | Education about FODMAP (fermentable oligosaccharides, disaccharides, monosaccharides and polyols) exclusion diet |
|  | Dietary Support Services | CTV3 | XaBSz | Refer to dietician |
|  | Dietary Support Services | CTV3 | XaIIa | Referral to dietician declined |
|  | Dietary Support Services | CTV3 | XaKHf | Health education - nutrition |
|  | Dietary Support Services | CTV3 | XaLoI | In-house dietetics 1st appt |
|  | Dietary Support Services | CTV3 | XaLoK | In-house dietetics f/up appt |
|  | Dietary Support Services | CTV3 | XaQaU | Lifestyle advice regard diet |
|  | Dietary Support Services | CTV3 | XaX5m | Intervention for risk to health associated with overweight and obesity, advice about diet and physical activity, consider drugs |
|  | Dietary Support Services | CTV3 | XaZc6 | Dietary education for eat out |
|  | Dietary Support Services | CTV3 | XE0i1 | Patient advised about weight-reducing diet |
|  | Dietary Support Services | CTV3 | XE0i2 | Pt advise re lactose-free diet |
|  | Dietary Support Services | CTV3 | ZV653 | [V]Dietary counselling |
|  | Dietary Support Services | Readv2 | 66CS. | Intervention for risk to health associated with overweight and obesity, advice about diet and physical activity, consider drugs |
|  | Dietary Support Services | Readv2 | 66CT. | Intervention for risk to health associated with overweight and obesity, advice about diet and physical activity, consider drugs, consider surgery |
|  | Dietary Support Services | Readv2 | 6798. | Health ed. - exercise |
|  | Dietary Support Services | Readv2 | 6799. | Health ed. - diet |
|  | Dietary Support Services | Readv2 | 67H7. | Lifestyle advice regard diet |
|  | Dietary Support Services | Readv2 | 67Ik. | Patient advise about nutrition |
|  | Dietary Support Services | Readv2 | 8CA40 | Pt advised re wt reducing diet |
|  | Dietary Support Services | Readv2 | 8CA42 | Pt advised re gluten free diet |
|  | Dietary Support Services | Readv2 | 8CA43 | Patient advised about phenylalanine-free diet |
|  | Dietary Support Services | Readv2 | 8CA44 | Pt advised re high fibre diet |
|  | Dietary Support Services | Readv2 | 8CA45 | Pt advised re low residue diet |
|  | Dietary Support Services | Readv2 | 8CA46 | Pt advised re low fat diet |
|  | Dietary Support Services | Readv2 | 8CA47 | Pt advis-low cholesterol diet |
|  | Dietary Support Services | Readv2 | 8CA48 | Pt advised re low salt diet |
|  | Dietary Support Services | Readv2 | 8CA49 | Pt advised - lactose free diet |
|  | Dietary Support Services | Readv2 | 8CA4A | Pt advis-low carbohydrate diet |
|  | Dietary Support Services | Readv2 | 8CA4B | Pt advised re low protein diet |
|  | Dietary Support Services | Readv2 | 8CA4C | Pt advised - high protein diet |
|  | Dietary Support Services | Readv2 | 8CA4D | Pt advised re milk free diet |
|  | Dietary Support Services | Readv2 | 8CA4E | Pt advised re egg free diet |
|  | Dietary Support Services | Readv2 | 8CA4F | Pt advised re vegetarian diet |
|  | Dietary Support Services | Readv2 | 8CA4G | Pt advised re vegan diet |
|  | Dietary Support Services | Readv2 | 8CA4R | Fluid restriction advice |
|  | Dietary Support Services | Readv2 | 8CA4S | Diet educatn for food allergy |
|  | Dietary Support Services | Readv2 | 8CA4X | Recommendation to take ONS |
|  | Dietary Support Services | Readv2 | 8CA4Y | Recommend take home made ONS |
|  | Dietary Support Services | Readv2 | 8CE4. | Diet leaflet given |
|  | Dietary Support Services | Readv2 | 8CJ.. | Feeding and dietary regimes |
|  | Dietary Support Services | Readv2 | 8H76. | Refer to dietician |
|  | Dietary Support Services | Readv2 | 8H760 | Referral to commun dietician |
|  | Dietary Support Services | Readv2 | 9N6s. | Ref by hosp-based dietitian |
|  | Dietary Support Services | Readv2 | 9N6t. | Refer by comm-based dietitian |
|  | Dietary Support Services | Readv2 | 9NNF. | Under care of dietitian |
|  | Dietary Support Services | Readv2 | ZV653 | [V]Diet surveillance/counsel. |
| 2 | Domestic Violence Support Services | CTV3 | 67Ia. | Advice about domestic violence |
|  | Domestic Violence Support Services | CTV3 | 8Hl7. | Refer domest violence advocate |
| 1 | Education Support Services | CTV3 | 8Hj.. | Referral to education service |
| 20 | Employment Support Services | CTV3 | 13JN. | Job training needed |
|  | Employment Support Services | CTV3 | 13OD. | Employment support allowance |
|  | Employment Support Services | CTV3 | 13R.. | Unemployment benefits |
|  | Employment Support Services | CTV3 | 13R1. | Unemployment benefit |
|  | Employment Support Services | CTV3 | 13RZ. | Unemployment benefit NOS |
|  | Employment Support Services | CTV3 | 67N0. | Employment counselling |
|  | Employment Support Services | CTV3 | 8F45. | Vocational re-education |
|  | Employment Support Services | CTV3 | 9N6i. | Referral by employment service |
|  | Employment Support Services | CTV3 | Ua0Tb | Registered with job centre |
|  | Employment Support Services | CTV3 | Ua0Td | Attends job club |
|  | Employment Support Services | CTV3 | Ua0Te | Regstd wth dsblmt rstlmnt ofcr |
|  | Employment Support Services | CTV3 | Ua0TK | On pathway to employmnt scheme |
|  | Employment Support Services | CTV3 | Ub0sw | Employment retraining |
|  | Employment Support Services | CTV3 | X71Fu | Blind rehab - employmt placemt |
|  | Employment Support Services | CTV3 | XaAyh | On volunt sect employm scheme |
|  | Employment Support Services | CTV3 | XaECA | Unemployment counselling |
|  | Employment Support Services | CTV3 | XaXvu | Referral fr employment support |
|  | Employment Support Services | CTV3 | XaZHh | Employmnt support allow status |
|  | Employment Support Services | CTV3 | XaZYB | Employment support indicated |
|  | Employment Support Services | CTV3 | XM1aE | Job training needed |
| 4 | Finance Support Services | CTV3 | 13U.. | Low income benefits |
|  | Finance Support Services | CTV3 | 13UZ. | Low income benefits NOS |
|  | Finance Support Services | CTV3 | Xag09 | Prov wrtn info financial spprt |
|  | Finance Support Services | CTV3 | Xag0D | Prov writn info social support |
| 15 | General Lifestyle Support Services | CTV3 | .678. | Health education - general |
|  | General Lifestyle Support Services | CTV3 | .6781 | Health education offered |
|  | General Lifestyle Support Services | CTV3 | .6783 | Health education given |
|  | General Lifestyle Support Services | CTV3 | 679P. | Health ed - weight management |
|  | General Lifestyle Support Services | CTV3 | Ua02p | Advice relat health-rel behav |
|  | General Lifestyle Support Services | CTV3 | Ua04e | Teaching how to avoid problem |
|  | General Lifestyle Support Services | CTV3 | XaEFY | Lifestyle counselling |
|  | General Lifestyle Support Services | CTV3 | XaKHd | Health ed - weight management |
|  | General Lifestyle Support Services | CTV3 | Xa0iG | Health education |
|  | General Lifestyle Support Services | CTV3 | Xa1dc | Counselling/health education |
|  | General Lifestyle Support Services | CTV3 | ZV654 | [V]Health education |
|  | General Lifestyle Support Services | Readv2 | 678.. | Health education - general |
|  | General Lifestyle Support Services | Readv2 | 6781. | Health education offered |
|  | General Lifestyle Support Services | Readv2 | 6783. | Health education given |
|  | General Lifestyle Support Services | Readv2 | ZV654 | [V]Health education |
| 27 | General Social Support Services | CTV3 | 8O85. | Social support |
|  | General Social Support Services | CTV3 | Ua0Vj | Neighbourhood care support |
|  | General Social Support Services | CTV3 | Ua0Vk | Support from caring community |
|  | General Social Support Services | CTV3 | Ua0Vl | Support from local milkman |
|  | General Social Support Services | CTV3 | Ua0Vm | Support from local postman |
|  | General Social Support Services | CTV3 | XaaUc | Ref volntry suport servc carer |
|  | General Social Support Services | CTV3 | XaJQr | Has cont with multi supp agenc |
|  | General Social Support Services | CTV3 | XM1Zu | Self-help group support |
|  | General Social Support Services | Readv2 | 8Hk7. | Referred for health coaching (finding) |
|  | General Social Support Services | CTV3 | .8O3. | Long term social support |
|  | General Social Support Services | CTV3 | 13Y.. | Self-help group support |
|  | General Social Support Services | CTV3 | 8BJ2. | Supportive care |
|  | General Social Support Services | CTV3 | 8HHN0 | Ref volntry suport servc carer |
|  | General Social Support Services | CTV3 | 8O3.. | Long term social support |
|  | General Social Support Services | CTV3 | 8O8.. | Support |
|  | General Social Support Services | CTV3 | 9N2o. | Seen by health support worker |
|  | General Social Support Services | CTV3 | 9Ngc. | Has healthcare support worker |
|  | General Social Support Services | CTV3 | 9NN3. | Has support worker |
|  | General Social Support Services | Readv2 | 13Y.. | Self-help group support |
|  | General Social Support Services | Readv2 | 8BJ2. | Supportive care |
|  | General Social Support Services | Readv2 | 8HHN0 | Referral to voluntary support service for carers |
|  | General Social Support Services | Readv2 | 8O3.. | Long term social support |
|  | General Social Support Services | Readv2 | 8O8.. | Support |
|  | General Social Support Services | Readv2 | 8O85. | Social support |
|  | General Social Support Services | Readv2 | 9N2o. | Seen by health support worker |
|  | General Social Support Services | Readv2 | 9Ngc. | Has healthcare support worker |
|  | General Social Support Services | Readv2 | 9NN3. | Has support worker |
| 19 | Home-based Support Services | CTV3 | 13G6. | Home help |
|  | Home-based Support Services | CTV3 | 13G61 | Home help attends |
|  | Home-based Support Services | CTV3 | 13G63 | Home help organised |
|  | Home-based Support Services | CTV3 | 8HH2. | Arrange home help |
|  | Home-based Support Services | CTV3 | Ua1Ne | Home support |
|  | Home-based Support Services | CTV3 | Ua1TP | Accident preventn in the home |
|  | Home-based Support Services | CTV3 | .13C4 | Needs walking aid in home |
|  | Home-based Support Services | CTV3 | 133c. | Hospital at home patient |
|  | Home-based Support Services | CTV3 | 13C4. | Needs walking aid in home |
|  | Home-based Support Services | CTV3 | 13G62 | Home help requested |
|  | Home-based Support Services | Readv2 | 133c. | Hospital at home patient |
|  | Home-based Support Services | Readv2 | 13C4. | Needs walking aid in home |
|  | Home-based Support Services | Readv2 | 13CX. | Moving and handling equipment available at home |
|  | Home-based Support Services | Readv2 | 13G6. | Home help |
|  | Home-based Support Services | Readv2 | 13G61 | Home help attends |
|  | Home-based Support Services | Readv2 | 13G62 | Home help requested |
|  | Home-based Support Services | Readv2 | 13G63 | Home help organised |
|  | Home-based Support Services | Readv2 | 13G64 | Home help needed |
|  | Home-based Support Services | Readv2 | 8HH2. | Arrange home help |
| 25 | Housing Support Services | CTV3 | .9k6.. | Homeless ESA |
|  | Housing Support Services | CTV3 | 13U3. | Housing benefit |
|  | Housing Support Services | CTV3 | 67IB. | Home safety advice |
|  | Housing Support Services | CTV3 | 8H7y. | Referral to housing department |
|  | Housing Support Services | CTV3 | 8Hk9. | Referral to home safety servic |
|  | Housing Support Services | CTV3 | 9k60. | Homeless - enh serv completed |
|  | Housing Support Services | CTV3 | 9Ngr. | Undr cre of hmlss advccy srvce |
|  | Housing Support Services | CTV3 | 9R31. | Letter written to housing |
|  | Housing Support Services | CTV3 | 9R32. | On urgent housing list |
|  | Housing Support Services | CTV3 | 9R33. | On housing list |
|  | Housing Support Services | CTV3 | 9R34. | Awaiting housing improvement |
|  | Housing Support Services | CTV3 | 9R35. | Referred for accomodation: [++ |
|  | Housing Support Services | CTV3 | 9R37. | Housing problem solved |
|  | Housing Support Services | CTV3 | Ua0fJ | Hostel for the homeless |
|  | Housing Support Services | CTV3 | XaAq1 | Discharge to sheltered housing |
|  | Housing Support Services | CTV3 | XacTB | Request for minor hous adaptat |
|  | Housing Support Services | CTV3 | XacTC | Minor hous adaptation complete |
|  | Housing Support Services | CTV3 | XacTD | Major hous adaptation complete |
|  | Housing Support Services | CTV3 | XacTF | Recommend major adapt DFG appl |
|  | Housing Support Services | CTV3 | XadHB | Housing educ guidance counsell |
|  | Housing Support Services | CTV3 | XagM0 | Signpost to housing supprt srv |
|  | Housing Support Services | CTV3 | XagnG | Ref to homeless advocacy srvce |
|  | Housing Support Services | CTV3 | XagnO | Ref to homeless advc srv dclnd |
|  | Housing Support Services | CTV3 | Xagzf | Prov of com otrch cre fr homls |
|  | Housing Support Services | CTV3 | XaJQg | Referral by housing services |
| 17 | Mental Health Services | CTV3 | Ua1Nf | Emotional support |
|  | Mental Health Services | CTV3 | XaIOv | Men heal supp gr - staff facil |
|  | Mental Health Services | CTV3 | XaIOx | Mental health carers' support |
|  | Mental Health Services | CTV3 | XaIP3 | Ment healt supp - no facilitat |
|  | Mental Health Services | CTV3 | XaIpc | Emotion + psychosoc supp + adv |
|  | Mental Health Services | CTV3 | XaIWx | MHsup gr stf fac - 1-2 cont/wk |
|  | Mental Health Services | CTV3 | XaIWy | MH sup gr stf fac - 1-3 cont/m |
|  | Mental Health Services | CTV3 | XaIWz | MH sup gr stf fac - 1-3 cont/m |
|  | Mental Health Services | CTV3 | XaIX0 | MH sup no facilt - 1-2 cont/wk |
|  | Mental Health Services | CTV3 | XaIXN | MH carers up - 3-5 contact/wk |
|  | Mental Health Services | CTV3 | XaIXP | MH carers' suprt-1-2 contcts/wk |
|  | Mental Health Services | CTV3 | XaIXQ | MH carers' suprt-1-3 contcts/mon |
|  | Mental Health Services | CTV3 | XaIXR | MH carers' suprt-<1contact/mon |
|  | Mental Health Services | CTV3 | XaIXY | MH sup no facil - 1-3 cont/mth |
|  | Mental Health Services | CTV3 | XaIXZ | MH sup no facil - <1 conct/mth |
|  | Mental Health Services | CTV3 | .8O82 | Emotion + psychosoc supp + adv |
|  | Mental Health Services | Readv2 | 8O82. | Emotion + psychosoc supp + adv |
| 21 | Support Services for Other Conditions | CTV3 | XaPvz | Refer to YISP |
|  | Support Services for Other Conditions | CTV3 | XaPzS | Appli Macmillan ca spprt grant |
|  | Support Services for Other Conditions | CTV3 | XaQGt | Refer dysphasia support servce |
|  | Support Services for Other Conditions | CTV3 | .8HH6 | Referral to Macmillan nurse |
|  | Support Services for Other Conditions | CTV3 | .9NNS | Under care of Macmillan nurse |
|  | Support Services for Other Conditions | CTV3 | 8H761 | Referrl to Macmillan dietitian |
|  | Support Services for Other Conditions | CTV3 | 8H770 | Referral to Macmillan physio |
|  | Support Services for Other Conditions | CTV3 | 8H7J0 | Referral to Macmillan OT |
|  | Support Services for Other Conditions | CTV3 | 8HH6. | Referral to Macmillan nurse |
|  | Support Services for Other Conditions | CTV3 | 9NNS. | Under care of Macmillan nurse |
|  | Support Services for Other Conditions | CTV3 | XaAQb | Under care of Macmillan nurse |
|  | Support Services for Other Conditions | CTV3 | XaATy | Seen by Macmillan nurse |
|  | Support Services for Other Conditions | CTV3 | Xabws | Referral to Macmillan physio |
|  | Support Services for Other Conditions | CTV3 | XacGv | Referrl to Macmillan dietitian |
|  | Support Services for Other Conditions | CTV3 | XacGw | Referral to Macmillan OT |
|  | Support Services for Other Conditions | CTV3 | XafEn | Sgnpstg Macmillan Cancer Sppt |
|  | Support Services for Other Conditions | Readv2 | 8H761 | Referrl to Macmillan dietitian |
|  | Support Services for Other Conditions | Readv2 | 8H770 | Referral to Macmillan physio |
|  | Support Services for Other Conditions | Readv2 | 8H7J0 | Referral to Macmillan OT |
|  | Support Services for Other Conditions | Readv2 | 8HH6. | Referral to Macmillan nurse |
|  | Support Services for Other Conditions | Readv2 | 9NNS. | Under care of Macmillan nurse |
| 139 | Parental Support Services | CTV3 | 13S.. | Pregnancy benfits |
|  | Parental Support Services | CTV3 | 13SZ. | Pregnancy benfits NOS |
|  | Parental Support Services | CTV3 | 13T.. | Parent's benefits |
|  | Parental Support Services | CTV3 | 13TZ. | Parent's benefits NOS |
|  | Parental Support Services | CTV3 | Xab6S | Attends parent support group |
|  | Parental Support Services | CTV3 | XaIBF | Vulnerable family support |
|  | Parental Support Services | CTV3 | XaLRb | Health education - fam support |
|  | Parental Support Services | CTV3 | XaLRh | Health ed. - mat & child bnfit |
|  | Parental Support Services | CTV3 | XaYX8 | Referral to postnatal group |
|  | Parental Support Services | Readv2 | 8Hl8. | Referral to breast feeding peer support service (procedure) |
|  | Parental Support Services | CTV3 | .13S. | Benefits - pregnancy |
|  | Parental Support Services | CTV3 | .13T. | Parent's benefits |
|  | Parental Support Services | CTV3 | .13T3 | One parent benefit |
|  | Parental Support Services | CTV3 | .13TZ | Parents benefit NOS |
|  | Parental Support Services | CTV3 | .6722 | Family counselled |
|  | Parental Support Services | CTV3 | .679N | Health education - parenting |
|  | Parental Support Services | CTV3 | .67A. | Pregnancy advice |
|  | Parental Support Services | CTV3 | .67A2 | Diet in pregnancy advice |
|  | Parental Support Services | CTV3 | .67A3 | Pregnancy smoking advice |
|  | Parental Support Services | CTV3 | .67A4 | Pregnancy exercise advice |
|  | Parental Support Services | CTV3 | .67A5 | Pregnancy alcohol advice |
|  | Parental Support Services | CTV3 | .67A6 | Drugs in pregnancy advice |
|  | Parental Support Services | CTV3 | .67A7 | Pregnancy dental advice |
|  | Parental Support Services | CTV3 | .67AB | Pregnancy prescription exemption advice |
|  | Parental Support Services | CTV3 | .67C. | Postnatal support group |
|  | Parental Support Services | CTV3 | .6G0. | Postnatal counselling |
|  | Parental Support Services | CTV3 | .6G00 | Postnatal depression counsel |
|  | Parental Support Services | CTV3 | .8C1H | Breast feeding education |
|  | Parental Support Services | CTV3 | .8C91 | Parent reassured |
|  | Parental Support Services | CTV3 | .8CI. | Had a chat to parent |
|  | Parental Support Services | CTV3 | .8G21 | Family psychotherapy |
|  | Parental Support Services | CTV3 | .8Hl8 | Referral to breast feeding peer support service |
|  | Parental Support Services | CTV3 | .8HTA | Referral to postnatal clinic |
|  | Parental Support Services | CTV3 | .8O4. | Vulnerable family support |
|  | Parental Support Services | CTV3 | .8O80 | Parental support |
|  | Parental Support Services | CTV3 | 13T3. | One parent benefit |
|  | Parental Support Services | CTV3 | 1a1.. | Attends parent support group |
|  | Parental Support Services | CTV3 | 62D4. | Parent craft class attended |
|  | Parental Support Services | CTV3 | 62D5. | Parent craft -individual class |
|  | Parental Support Services | CTV3 | 62D6. | Parent craft - group class |
|  | Parental Support Services | CTV3 | 62DZ. | Parent craft class NOS |
|  | Parental Support Services | CTV3 | 6722. | Family counselled |
|  | Parental Support Services | CTV3 | 677G. | Family counselling |
|  | Parental Support Services | CTV3 | 67910 | Health ed. - parental smoking |
|  | Parental Support Services | CTV3 | 679I. | Health ed - infant massage |
|  | Parental Support Services | CTV3 | 679N. | Health education - parenting |
|  | Parental Support Services | CTV3 | 67C.. | Postnatal support group |
|  | Parental Support Services | CTV3 | 6G0.. | Postnatal counselling |
|  | Parental Support Services | CTV3 | 6G00. | Postnatal depression counsel |
|  | Parental Support Services | CTV3 | 8C1H. | Breast feeding education |
|  | Parental Support Services | CTV3 | 8C91. | Parental reassurance |
|  | Parental Support Services | CTV3 | 8CI.. | Had a chat to parent |
|  | Parental Support Services | CTV3 | 8Cp.. | Triple P - Positive Parenting Programme |
|  | Parental Support Services | CTV3 | 8Cv.. | Mellow Parenting programme |
|  | Parental Support Services | CTV3 | 8Cx0. | Discussion about family wellbeing |
|  | Parental Support Services | CTV3 | 8G102 | Behavioural parent training |
|  | Parental Support Services | CTV3 | 8G130 | CBT parenting programme |
|  | Parental Support Services | CTV3 | 8G21. | Family psychotherapy |
|  | Parental Support Services | CTV3 | 8G210 | Functional Family Therapy |
|  | Parental Support Services | CTV3 | 8G22. | Family intervention for substance misuse |
|  | Parental Support Services | CTV3 | 8GJ.. | Parent-infant psychotherapy |
|  | Parental Support Services | CTV3 | 8Hl8. | Referral to breast feeding peer support service |
|  | Parental Support Services | CTV3 | 8HTA. | Referral to postnatal clinic |
|  | Parental Support Services | CTV3 | 8O4.. | Vulnerable family support |
|  | Parental Support Services | CTV3 | 8O84. | Family support |
|  | Parental Support Services | CTV3 | 8T0a. | Referral to family nurse partnership team |
|  | Parental Support Services | CTV3 | 9NFb. | Health visitor 3 to 4 month contact with family |
|  | Parental Support Services | CTV3 | 9NFb0 | Health visitor 3 to 4 month contact with family declined |
|  | Parental Support Services | CTV3 | 9NFb3 | Health visitor 3 to 4 month contact with family completed by other healthcare provider |
|  | Parental Support Services | CTV3 | 9Nh3. | Under care of family nurse partnership team |
|  | Parental Support Services | CTV3 | 9NlZ. | Seen by family therapist |
|  | Parental Support Services | CTV3 | 9Nt8. | Seen by member of family nurse partnership team |
|  | Parental Support Services | CTV3 | Ub0rQ | Parenting skills training |
|  | Parental Support Services | CTV3 | X71FV | Breast feeding education |
|  | Parental Support Services | CTV3 | X71Fx | Parental support |
|  | Parental Support Services | CTV3 | XaaU9 | Parent-infant psychotherapy |
|  | Parental Support Services | CTV3 | XaaWs | Behavioural parent training |
|  | Parental Support Services | CTV3 | XaaZh | Postnatal listening visit |
|  | Parental Support Services | CTV3 | XabtP | Mellow Parenting programme |
|  | Parental Support Services | CTV3 | XaEK0 | Had a chat to parent |
|  | Parental Support Services | CTV3 | XafGb | Responsive parenting intervention |
|  | Parental Support Services | CTV3 | XaIpA | Postnatal counselling |
|  | Parental Support Services | CTV3 | XaIpB | Postnatal depression counsel |
|  | Parental Support Services | CTV3 | XaIqA | Health ed - infant massage |
|  | Parental Support Services | CTV3 | XaKCD | Health education - parenting |
|  | Parental Support Services | CTV3 | XaLRi | Health ed. - parental smoking |
|  | Parental Support Services | CTV3 | XaPEQ | Educatn parent to support play |
|  | Parental Support Services | CTV3 | XaPxs | Assessment parenting capacity |
|  | Parental Support Services | CTV3 | XaXOv | Helth eductn - sibling rivalry |
|  | Parental Support Services | CTV3 | XaXQ4 | Parent educn abou child health |
|  | Parental Support Services | CTV3 | XaYgS | Ref to postnatal depression gp |
|  | Parental Support Services | CTV3 | XE0tV | Parent's benefits |
|  | Parental Support Services | CTV3 | Y79uI | Parent craft classes |
|  | Parental Support Services | CTV3 | Y79uJ | Parent craft classes offered |
|  | Parental Support Services | CTV3 | Y79uL | Parent craft not wanted |
|  | Parental Support Services | Readv2 | 13S.. | Pregnancy benefits |
|  | Parental Support Services | Readv2 | 1a1.. | Attends parent support group |
|  | Parental Support Services | Readv2 | 62D.. | Parent craft classes |
|  | Parental Support Services | Readv2 | 62D1. | Parent craft classes offered |
|  | Parental Support Services | Readv2 | 62D3. | Parent craft not wanted |
|  | Parental Support Services | Readv2 | 62D4. | Parent craft class attended |
|  | Parental Support Services | Readv2 | 62D5. | Parent craft -individual class |
|  | Parental Support Services | Readv2 | 62D6. | Parent craft - group class |
|  | Parental Support Services | Readv2 | 6722. | Family counselled |
|  | Parental Support Services | Readv2 | 677G. | Family counselling |
|  | Parental Support Services | Readv2 | 67910 | Health ed. - parental smoking |
|  | Parental Support Services | Readv2 | 679N. | Health education - parenting |
|  | Parental Support Services | Readv2 | 679N0 | Parenting skills training |
|  | Parental Support Services | Readv2 | 67A.. | Pregnancy advice |
|  | Parental Support Services | Readv2 | 67A2. | Diet in pregnancy advice |
|  | Parental Support Services | Readv2 | 67A3. | Pregnancy smoking advice |
|  | Parental Support Services | Readv2 | 67A4. | Pregnancy exercise advice |
|  | Parental Support Services | Readv2 | 67A5. | Pregnancy alcohol advice |
|  | Parental Support Services | Readv2 | 67A6. | Drugs in pregnancy advice |
|  | Parental Support Services | Readv2 | 67A7. | Pregnancy dental advice |
|  | Parental Support Services | Readv2 | 67AB. | Pregnancy prescription exemption advice |
|  | Parental Support Services | Readv2 | 67C.. | Postnatal support group |
|  | Parental Support Services | Readv2 | 6G0.. | Postnatal counselling |
|  | Parental Support Services | Readv2 | 6G00. | Postnatal depression counselling |
|  | Parental Support Services | Readv2 | 8C1H. | Breast feeding education |
|  | Parental Support Services | Readv2 | 8Cx0. | Discussion abt family wllbeing |
|  | Parental Support Services | Readv2 | 8Cx00 | Family wellbeing discussion about mental health |
|  | Parental Support Services | Readv2 | 8Cx01 | Family wellbeing discussion about drugs |
|  | Parental Support Services | Readv2 | 8Cx02 | Family wellbeing discussion about alcohol |
|  | Parental Support Services | READV2 | 8G102 | Behavioural parent training |
|  | Parental Support Services | Readv2 | 8G21. | Family therapy |
|  | Parental Support Services | Readv2 | 8G210 | Functional Family Therapy |
|  | Parental Support Services | Readv2 | 8G22. | Family intervention for substance misuse |
|  | Parental Support Services | Readv2 | 8HTA. | Referral to postnatal clinic |
|  | Parental Support Services | Readv2 | 8O4.. | Vulnerable family support |
|  | Parental Support Services | Readv2 | 8O80. | Parental support |
|  | Parental Support Services | Readv2 | 8O84. | Family support |
|  | Parental Support Services | Readv2 | 8T0a. | Referral to family nurse partnership team |
|  | Parental Support Services | Readv2 | 9NFb. | Health visitor 3 to 4 month contact with family |
|  | Parental Support Services | Readv2 | 9NFb0 | Health visitor 3 to 4 month contact with family declined |
|  | Parental Support Services | Readv2 | 9NFb3 | Health visitor 3 to 4 month contact with family completed by other healthcare provider |
|  | Parental Support Services | Readv2 | 9Nh3. | Under care of family nurse partnership team |
|  | Parental Support Services | Readv2 | 9NlZ. | Seen by family therapist |
|  | Parental Support Services | Readv2 | 9Nt8. | Seen by member of family nurse partnership team |
| 89 | Physical Activity Management Services | CTV3 | 66CR. | Int rsk h ass ovwt ob diet phy |
|  | Physical Activity Management Services | CTV3 | 8CA52 | Educ abt chair based exer prog |
|  | Physical Activity Management Services | CTV3 | 8E79. | Home exercise programme |
|  | Physical Activity Management Services | CTV3 | 8H7s. | Referrl physical activ program |
|  | Physical Activity Management Services | Readv2 | 138S. | Declined referral to physical exercise programme |
|  | Physical Activity Management Services | Readv2 | 6798 | Health ed. - exercise |
|  | Physical Activity Management Services | Readv2 | 8BAH. | Exercise on prescription |
|  | Physical Activity Management Services | Readv2 | 8H7q0 | Referral for graded exercise therapy |
|  | Physical Activity Management Services | Readv2 | 8HBN. | Physical activity brief intervention follow-up |
|  | Physical Activity Management Services | Readv2 | 8HHc. | Referred for exercise programme |
|  | Physical Activity Management Services | Readv2 | 8HkX. | Referral to exercise on referral programme |
|  | Physical Activity Management Services | Readv2 | 8IAv. | Brief intervention for physical activity declined |
|  | Physical Activity Management Services | Readv2 | 8IAZ. | Referral for exercise on prescription declined |
|  | Physical Activity Management Services | Readv2 | 9NS01 | Referral for physical activity service offered |
|  | Physical Activity Management Services | Readv2 | 9Oq3. | Brief intervention for physical activity completed |
|  | Physical Activity Management Services | Readv2 | 9Oq5. | Brief intervention for physical activity offered |
|  | Physical Activity Management Services | Readv2 | 9Oq6. | Brief intervention for physical activity follow-up completed |
|  | Physical Activity Management Services | CTV3 | .138S | Decline ref phys exercise prog |
|  | Physical Activity Management Services | CTV3 | .13CQ | Physical activity target minimal exercise |
|  | Physical Activity Management Services | CTV3 | .13CR | Physical activity target light exercise |
|  | Physical Activity Management Services | CTV3 | .13CT | Physical activity target strenuous exercise |
|  | Physical Activity Management Services | CTV3 | .6798 | Health education - exercise |
|  | Physical Activity Management Services | CTV3 | .67H2 | Lifestyle adv re exercise |
|  | Physical Activity Management Services | CTV3 | .8BAH | Exercise on prescription |
|  | Physical Activity Management Services | CTV3 | .8CA5 | Patient advised re exercise |
|  | Physical Activity Management Services | CTV3 | .8E79 | Home exercise programme |
|  | Physical Activity Management Services | CTV3 | .8E7A | Group exercise programme |
|  | Physical Activity Management Services | CTV3 | .8E7B | Graded exercise therapy |
|  | Physical Activity Management Services | CTV3 | .8H7q | Referral for exercise therapy |
|  | Physical Activity Management Services | CTV3 | .8H7s | Referrl physical activ program |
|  | Physical Activity Management Services | CTV3 | .8HHc | Referred for exercise programm |
|  | Physical Activity Management Services | CTV3 | .9Oq3 | Brief intervention for physical activity completed |
|  | Physical Activity Management Services | CTV3 | 138S. | Decline ref phys exercise prog |
|  | Physical Activity Management Services | CTV3 | 6798. | Health education - exercise |
|  | Physical Activity Management Services | CTV3 | 67H2. | Lifestyle adv re exercise |
|  | Physical Activity Management Services | CTV3 | 8BAH. | Exercise on prescription |
|  | Physical Activity Management Services | CTV3 | 8CA5. | Patient advised re exercise |
|  | Physical Activity Management Services | CTV3 | 8CA50 | Advice about aerobic exercise |
|  | Physical Activity Management Services | CTV3 | 8CA51 | Advic abt muscle strength exer |
|  | Physical Activity Management Services | CTV3 | 8E7A. | Group exercise programme |
|  | Physical Activity Management Services | CTV3 | 8E7B. | Graded exercise therapy |
|  | Physical Activity Management Services | CTV3 | 8H7q. | Referral for exercise therapy |
|  | Physical Activity Management Services | CTV3 | 8H7q0 | Referral graded exercise thrpy |
|  | Physical Activity Management Services | CTV3 | 8HBN. | Physical activity brief intervention follow-up |
|  | Physical Activity Management Services | CTV3 | 8HHc. | Referred for exercise programm |
|  | Physical Activity Management Services | CTV3 | 8HkX. | Referral to exercise on referral programme |
|  | Physical Activity Management Services | CTV3 | 8IAv. | Brief intervention for physical activity declined |
|  | Physical Activity Management Services | CTV3 | 8IAZ. | Referral for exercise on prescription declined |
|  | Physical Activity Management Services | CTV3 | 9NS01 | Referral for physical activity service offered |
|  | Physical Activity Management Services | CTV3 | 9Oq3. | Brief intervention for physical activity completed |
|  | Physical Activity Management Services | CTV3 | 9Oq5. | Brief intervention for physical activity offered |
|  | Physical Activity Management Services | CTV3 | 9Oq6. | Brief intervention for physical activity follow-up completed |
|  | Physical Activity Management Services | CTV3 | Ub01e | Dietary advice for endurance exercise |
|  | Physical Activity Management Services | CTV3 | Xa1dN | Advice to exercise |
|  | Physical Activity Management Services | CTV3 | Xa9zF | Advice about exercise |
|  | Physical Activity Management Services | CTV3 | XaA0G | Giving encouragement to exercise |
|  | Physical Activity Management Services | CTV3 | XaA0R | Reassuring about exercise |
|  | Physical Activity Management Services | CTV3 | XaA0U | Assisting with exercise |
|  | Physical Activity Management Services | CTV3 | XabFV | Education about chair based exercise programme |
|  | Physical Activity Management Services | CTV3 | XaCmH | Exercise on prescription |
|  | Physical Activity Management Services | CTV3 | XagLq | Signposting to community exercise group |
|  | Physical Activity Management Services | CTV3 | XaIPu | Referral for exercise therapy |
|  | Physical Activity Management Services | CTV3 | XaItq | Group exercise programme |
|  | Physical Activity Management Services | CTV3 | XaJIt | Lifestyle adv re exercise |
|  | Physical Activity Management Services | CTV3 | XaJPL | Physical activity target minimal exercise |
|  | Physical Activity Management Services | CTV3 | XaJPN | Physical activity target strenuous exercise |
|  | Physical Activity Management Services | CTV3 | XaJPO | Physical activity target light exercise |
|  | Physical Activity Management Services | CTV3 | XaJPP | Physical activity target moderate exercise |
|  | Physical Activity Management Services | CTV3 | XaKRq | Referred for exercise programm |
|  | Physical Activity Management Services | CTV3 | XaL1X | Decline ref phys exercise prog |
|  | Physical Activity Management Services | CTV3 | XaP45 | Exercise leaflet given |
|  | Physical Activity Management Services | CTV3 | XaPdD | Graded exercise therapy |
|  | Physical Activity Management Services | CTV3 | XaQiT | Refer exercise prescrip declin |
|  | Physical Activity Management Services | CTV3 | XaR5C | Referral graded exercise thrpy |
|  | Physical Activity Management Services | CTV3 | XaREh | Referral to exercise on referral programme |
|  | Physical Activity Management Services | CTV3 | XaX5l | Intervention for risk to health associated with overweight and obesity, advice about diet and physical activity |
|  | Physical Activity Management Services | CTV3 | XaYSS | Advice about aerobic exercise |
|  | Physical Activity Management Services | CTV3 | XaYST | Provision of advice about muscle strengthening exercise |
|  | Physical Activity Management Services | CTV3 | XM18T | Patient given exercise advice |
|  | Physical Activity Management Services | Readv2 | 13CQ. | Physical act targ min exercise |
|  | Physical Activity Management Services | Readv2 | 13CR. | Physical act targ light exer |
|  | Physical Activity Management Services | Readv2 | 13CS. | Physical act targ mod exercise |
|  | Physical Activity Management Services | Readv2 | 13CT. | Physical act targ stren exer |
|  | Physical Activity Management Services | Readv2 | 66CR. | Intervention for risk to health associated with overweight and obesity, advice about diet and physical activity |
|  | Physical Activity Management Services | Readv2 | 8CA52 | Educ abt chair based exer prog |
|  | Physical Activity Management Services | Readv2 | 8E79. | Home exercise programme |
|  | Physical Activity Management Services | Readv2 | 8E7A. | Group exercise programme |
|  | Physical Activity Management Services | Readv2 | 8E7B. | Graded exercise therapy |
|  | Physical Activity Management Services | Readv2 | 8H7s. | Referral to physical activity programme |
| 6 | Respiratory Support Services | CTV3 | 8Hlj. | Ref BLF breath easy supp group |
|  | Respiratory Support Services | CTV3 | XaIUX | Attends respirat support group |
|  | Respiratory Support Services | CTV3 | XaRFj | Health Education- asthma self management (procedure) |
|  | Respiratory Support Services | CTV3 | XaYfy | Referral to support group |
|  | Respiratory Support Services | CTV3 | XaZ2n | Ref BLF breath easy supp group |
|  | Respiratory Support Services | Readv2 | 745G2 | Respiratory health self management education (procedure) |
| 13 | Social Prescribing | Readv2 | 8BAf. | Social prescribing for mental health |
|  | Social Prescribing | Readv2 | 8IEp. | Social prescribing declined |
|  | Social Prescribing | Readv2 | 8T09. | Referral to social prescribing service |
|  | Social Prescribing | Readv2 | 9NSE. | Social prescribing offered |
|  | Social Prescribing | CTV3 | 8BAf. | Social prescrib mental health |
|  | Social Prescribing | CTV3 | 8IEp. | Social prescribing declined |
|  | Social Prescribing | CTV3 | 8T09. | Ref to social prescribing serv |
|  | Social Prescribing | CTV3 | 9NSE. | Social prescribing offered |
|  | Social Prescribing | CTV3 | XaaEA | Social prescribing offered |
|  | Social Prescribing | CTV3 | XaaEB | Social prescribing declined |
|  | Social Prescribing | CTV3 | XaaEC | Ref to social prescribing serv |
|  | Social Prescribing | CTV3 | XagOR | Signpost social prescrib serv |
|  | Social Prescribing | CTV3 | XaQvz | Social prescrib mental health |
| 9 | Stress Reduction Support Services | CTV3 | .67J. | Stress counselling |
|  | Stress Reduction Support Services | CTV3 | 9ON1. | Attends stress monitoring |
|  | Stress Reduction Support Services | CTV3 | 9ON2. | Refuses stress monitoring |
|  | Stress Reduction Support Services | CTV3 | Ub014 | Stress management |
|  | Stress Reduction Support Services | CTV3 | 67J.. | Stress counselling |
|  | Stress Reduction Support Services | CTV3 | XaI8j | Stress counselling |
|  | Stress Reduction Support Services | Readv2 | 67J.. | Stress counselling |
|  | Stress Reduction Support Services | Readv2 | 9ON1. | Attends stress monitoring |
|  | Stress Reduction Support Services | Readv2 | 9ON2. | Refuses stress monitoring |
